# Taking Care of Time

**Published:** 2007

**Authors:** Cortney Davis

**Affiliations:** PO Box 678, Redding, CT 06896-0678 Email: cortneydav@aol.com, www.cortneydavis.com

*So take good care of time, therefore and how you spend it.*
-from “*The Cloud of Unknowing”*

Yesterday it was a thousand small coins
ringing in your pocket, your hand dipping in, scooping three
at a time, giving them away. Often you'd drop one
in the lush grass, unaware it was lost.

Spent, tarnished, it is irretrievable.

Today time comes to you in a different disguise:
a bold of fine silk, vermillion or blue, you measure it
like a woman preparing to sew.

Tomorrow, *watch out,* it comes as something else-
thunderstorm, slant rain, February blizzard that drives you inside.

Insomniac, you pace and curse
the blue glow of television, computer screen, radio.

Soon enough, time will come to you as you were once,
newly born and difficult to recognize. You could mistake it
for an elderly coughing man or a woman overrun with disease.

Do not stop your ears against its cry.

It will ask you to return any small change.

It will say, cherish every moment under the leaden sky.

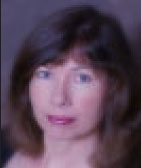

About the Poet

Cortney Davis, a nurse practitioner in women's health, is the author of three poetry collections, most recently “Leopold's Maneuvers,” winner of the Prairie Schooner Poetry Prize. A memoir about her work, “I Knew a Woman: the Experience of the Female Body,” was published by Random House. Her website is www.cortneydavis.com

